# Tat_BioV: tattoo ink exposure and biokinetics of selected tracers in a short-term clinical study of 24 subjects

**DOI:** 10.1007/s00204-025-03959-8

**Published:** 2025-01-31

**Authors:** Susanne Kochs, Sandra Schiewe, Milena Foerster, Kathrin Hillmann, Claudia Blankenstein, Martina C. Meinke, Josephine Kugler, David Kocovic, Andreas Luch, Ulrike Blume-Peytavi, Ines Schreiver

**Affiliations:** 1 Department of Chemical and Product Safety, German Federal Institute for Risk Assessment (BfR), Berlin, Germany; 2 International Agency for Research On Cancer (IARC), Environment and Lifestyle Epidemiology Branch, Lyon, France; 3 Department of Dermatology, Venereology and Allergology, Clinical Research Center for Hair and Skin Science, Charité-Universitätsmedizin Berlin, Corporate Member of Freie Universität Berlin and Humboldt Universität Zu Berlin, Berlin, Germany; 4 Department of Dermatology, Venereology and Allergology, Center of Experimental and Applied Cutaneous Physiology, Charité-Universitätsmedizin Berlin, Corporate Member of Freie Universität Berlin and Humboldt Universität Zu Berlin, Berlin, Germany; 5 Center for Inspection Supervision and Market Control, Institute for Medicines and Medical Devices of Montenegro, Podgorica, Montenegro

**Keywords:** Tattooing, Skin, Environmental exposure, Metabolism, Mass spectrometry, Clinical study

## Abstract

About one-fifth of people in industrialised countries are tattooed, potentially putting them at risk of exposure to possible carcinogenic or otherwise harmful substances. This study aims to determine the exposure to soluble tattoo ink ingredients and their excretion within 24 h after tattooing. In this clinical study, 24 subjects were tattooed with black or red tattoo ink to which the 3 tracer substances, potassium iodide, 4-aminobenzoic acid (PABA) and 2-phenoxyethanol (PEtOH), had been added to mimic known substances found in tattoo inks. Tracers and their metabolites were quantified in blood, urine, ink and consumables pre- and post-tattooing. Tattooed skin area was determined using picture analysis. PABA metabolism upon tattooing was compared to peroral administration. Skin fibroblasts and macrophages were tested in vitro for their ability to metabolise PABA. All tracers or their metabolites were identified in urine; iodide and the PABA metabolite 4-acetamidobenzoic acid (ACD) were identified in plasma. The worst-case scenario for systemic ink exposure was estimated to be 0.31 g ink per tattoo session (75th percentile). Peroral administration resulted in lower levels of ACD than tattooing. Fibroblasts and macrophages were capable of converting PABA into ACD. Our results are the first human in vivo data on soluble tattoo ink ingredients and suggest that the overall exposure might be lower than the estimates previously used for regulatory purposes. In addition, the first-pass effect by skin metabolism leads to an altered metabolite profile compared to oral exposure. Skin metabolism might also contribute to detoxification of certain carcinogenic substances through *N*-acetylation.

**Supplementary Information:**

The online version contains supplementary material available at 10.1007/s00204-025-03959-8.

## Introduction

The high prevalence of body tattooing and its facial equivalent, permanent make-up, has already raised health concerns for decades (McGovern 2005). Between 17 and 31.5% of all people across Europe and the United States are tattooed (Borkenhagen et al. 2019; Kluger et al. 2019). The European REACH (Registration, Evaluation, Authorisation and Restriction of Chemicals) restriction on tattoo and permanent make-up inks bans more than 4000 substances due to their hazard potential, thus making it the strictest regulation worldwide (European Parliament and European Council 2020). Substances with toxic potential found in tattoo inks include heavy metals (e.g. chromium, nickel, cobalt), primary aromatic amines (PAAs) and polycyclic aromatic hydrocarbons (PAHs), which may have allergic and/or carcinogenic potential (Agnello and Fontana 2015; Battistini et al. 2020; Forte et al. 2009; Lehner et al. 2011, 2014; Lim and Shin 2017; Regensburger et al. 2010). Whilst acute adverse reactions occur promptly after tattooing and are, therefore, relatively easy to correlate with them, long-term effects of tattoos, such as cancer and immunotoxicity, are not easily linked (Foerster et al. 2022). Exposure quantification is crucial to calculate safe limits and to assess population-wide risks associated with intentionally or non-intentionally added hazardous substances in tattoo inks.

During tattooing, insoluble pigments and soluble co-formulants are introduced into the dermal layer of the skin. Previous studies as well as clinical observations have shown that pigments can be transported to regional lymph nodes of tattooed individuals (Schreiver et al. 2017), indicating the possibility of further migration towards more distant organs. Since pigments remain in the body, long-term exposure has to be considered, whereas water soluble ingredients are likely to be metabolised and excreted rapidly. In this case, a short-term, acute exposure period can be assumed. Exposure through tattooing is determined by the amount of ink applied per surface area of the tattooed skin. Several studies were conducted to determine exposure levels, including weighing ex vivo skin before and after tattooing (Arbache et al. 2019) and quantifying pigments after tattooing of ex vivo skin (Engel et al. 2008). However, these data primarily refer to the initial pigment deposition rather than the soluble fraction administered, and they vary greatly (0.6 – 9.5 mg_pigment_/cm^2^) (Engel et al. 2008). Within the EU REACH Annex XV restriction report for tattoo and permanent make-up ink, an estimated exposure level of 14.36 mg_ink_/cm^2^ was calculated based on a study by Engel and colleagues (Engel et al. 2008; European Chemicals Agency 2017). For a tattooed area of 300 cm^2^, this would result in an equivalent to 4.308 g of ink per session. However, these were ex vivo generated data, and therefore do not consider the actual absorption of the soluble ink fraction into the body. Thus, their relevance for real-life exposure estimation remains elusive.

As tattooing is a unique kind of exposure route, comparison of metabolic profiles with peroral dosing—an administration route often used in toxicity studies—is of high interest. Skin metabolism was previously described as detoxifying for carcinogens such as PAAs (Grohmann et al. 2017). A different metabolic profile might, therefore, lead to increased or reduced adverse effects despite a similar systemic dose. Main driver of this detoxification is the *N*-acetylation of PAAs by keratinocytes (Grohmann et al. 2017). However, gene expression of *N*-acetyltransferase 1 (NAT1) was also found in fibroblasts (Bhaiya et al. 2006; Wiegand et al. 2014). Fibroblasts are the most common cell type in the dermis and tattoo pigments reside predominantly in fibroblasts and macrophages (Baranska et al. 2018; Strandt et al. 2020).

In the present study, we used an in vivo human quasi-experimental design to derive a reasonable worst-case exposure scenario that may be applied in future risk assessments. As most of the potentially harmful tattoo ink ingredients cannot be used in human experimental exposure studies due to their hazard potential, we added tracer substances (potassium iodide, 4-aminobenzoic acid (PABA), and 2-phenoxyethanol (PEtOH)) to tattoo inks, as hazard-free alternatives. To estimate the short-term exposure and biodistribution of tattoo inks, the tracers were quantified in blood, urine and consumable products in contact with ink of 24 subjects using previously validated analytical methods (Kochs et al. 2023). The tattooed body surface per session was derived by digital picture analysis. In addition, skin and peroral metabolism of PABA was investigated in vitro and in vivo, respectively.

## Methods

### Study design

The single-arm study was conducted at the Clinical Research Center for Hair and Skin Science, Charité-Universitätsmedizin Berlin (Berlin, Germany) from November 2021 to September 2022. A total of 24 subjects were tattooed by professional tattoo artists with different commercially available tattoo inks (14 black, 10 red) spiked with the tracers iodide, PABA and PEtOH. All tracers used were of pharmaceutical quality, i.e. according to European Pharmacopoeia (Ph. Eur.), or in form of a drug product. Due to the better solubility of the potassium salt (KPABA), POTABA capsules (Glenwood GmbH Pharmazeutische Erzeugnisse, Munich, Germany) were used in the case of PABA. PEtOH and potassium iodide (KI) were from Euro OTC Pharma GmbH (Bönen, Germany). The tracer concentrations were 30 mg/g_ink_ PABA (38.33 mg/g_ink_ KPABA), 5 mg/g_ink_ PEtOH and 4.44 mg/g_ink_ iodide (5.81 mg/g_ink_ KI). The concentrations of iodide and PABA were selected to be below levels used in drug products but above detection limits in blood and urine (Weidner et al. 2005). PEtOH was used at a concentration for preservation in accordance to regulation (EC) No 1223/2009 (European Parliament 2009). To avoid contamination of the ink, the spiking was carried out in a cytostatic workbench according to DIN 12980 and EU-GMP cleanroom classification grade A, which was located in a grade C cleanroom (cf. *Supplemental Material 1*). The tracers were selected based on their physico-chemical similarities to tattoo-associated substances, whilst having a low toxicological profile and also being used in drug products (Bührer et al. 2002; Kochs et al. 2023; Le Guen et al. 2007; Meyer et al. 2007; Nauman and Wolff 1993; Roy and Carrier 2008; Sharma et al. 2014). The tracer potassium iodide was used to determine the amount of ink applied to the dermis, since it is easy to track, not volatile at the used pH value and its quantification is not sensitive to metabolism or other processes. In medicine, iodide can be used in high concentrations to block the incorporation of the released radioactive iodine isotype by the thyroid hormone biosynthesis in case of nuclear incidents by saturating the responsible transport enzymes (Le Guen et al. 2007; Nauman and Wolff 1993; World Health Organisation 2011). As iodide is physiologically present in human blood, its background levels have to be considered in the measurements and by setting the tracer concentrations accordingly (Nasterlack et al. 2016). The second tracer used was PABA. The potassium salt of this compound is used as a dietary supplement (Sharma et al. 2014) and for the treatment of Peyronie’s disease (Weidner et al. 2005). However, concentrations used in this study were below levels used in drug products or dietary supplements (Sharma et al. 2014; Weidner et al. 2005). Here, it was chosen for its similarity to aryl compounds with an amino moiety, a common structure of PAAs. Furthermore, PABA was selected for its high urine retrieval rate of up to > 90% 24 h after administration, which makes it a reliable indicator of the completeness of 24 h-urine collection (Bingham and Cummings 1983; Chan et al. 1988; Jakobsen et al. 2012). The metabolism of PABA is well-documented with potential metabolites including 4-aminobenzoic acid glucuronide (PABA-GlcA), 4-acetamidobenzoic acid (ACD), 4-acetamidobenzoic acid glucuronide (ACD-GlcA), 4-aminohippuric acid (PAHA) and 4-acetamidohippuric acid (ACHA) (Chan et al. 1988). The third tracer was PEtOH, a preservative commonly used in cosmetic products and in some parenteral drugs (Bührer et al. 2002; Dréno et al. 2019), and sometimes also in tattoo inks.

Subjects and tattoo artists were recruited through flyers and social media. The inclusion criteria for subjects were: healthy, male, 18 – 45 years of age, 60 – 100 kg, autonomous wish for a tattoo (tattooed area ± 30%: black ~ 300 cm^2^, red ~ 100 cm^2^), and at least one already existing tattoo. The design of the tattoo and the tattoo artist were of subject’s choice. Exclusion criteria included health restrictions, intake of certain drugs or dietary supplements containing the tracers or that might otherwise affect the study, and unwillingness to follow the behavioural and dietary rules (*Supplemental Material 2*). Since PEtOH can be present in cosmetic products, subjects were instructed to use specified cosmetic products provided for the study period only.

Participation in the study involved 3–4 appointments for each subject. Preliminary examinations included parameters such as age, height, weight, body fat (three-site measurement technique according to Jackson and Pollock (Jackson and Pollock 1978)) and blood parameters to determine liver, kidney and thyroid function. Subjects were instructed on usage of urine canisters, dietary rules and provided with cosmetic products.

**Fig. 1:**
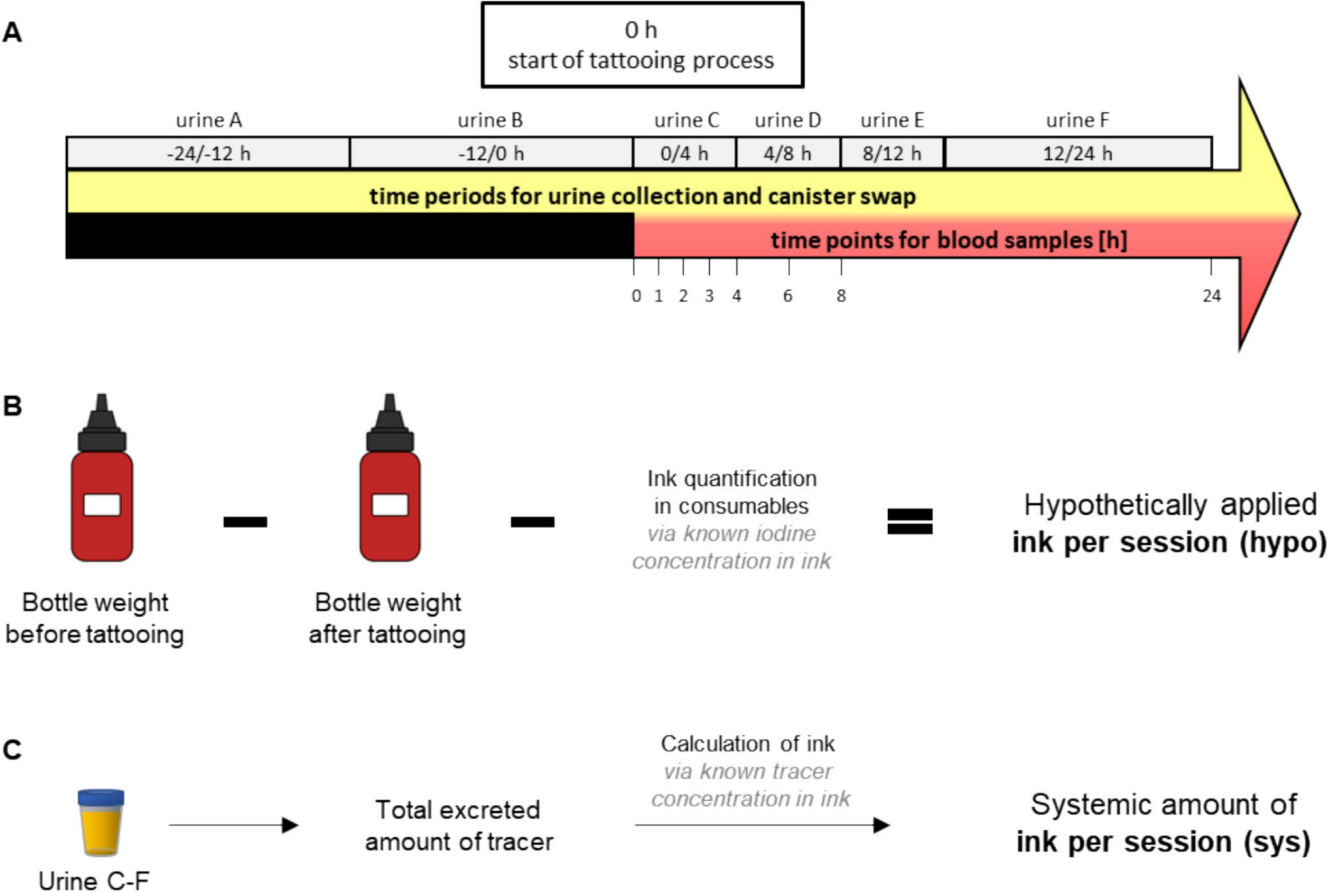
Study sampling and exposure calculation. **A** Overview of sampling times for blood and urine during the 48-h study period. **B** Calculation of the hypothetical ink amount used per session (hypo) based on ink weighing and iodine quantification in the consumables. **C** Calculation of the systemic ink amount per session (sys) calculated by excreted tracers in urine C-F. Background levels in urine A–B were subtracted

Urine was collected autonomously by subjects for 48 h with container changes at specific time intervals (Fig. 1). Blood samples were collected at specific time points (Fig. 1) at the study centre using 5 ml citrate–phosphate-dextrose-adenine (CPDA) blood collection tubes. During and at the end of the tattooing process, all consumables and items that had come into contact with the ink were collected in designated bags. The samples were then transported to the German Federal Institute for Risk Assessment (Bundesinstitut für Risikobewertung, BfR, Berlin, Germany) and analysed.

### Ethics approval

The study was approved by the ethics committee of the Charité—Universitätsmedizin Berlin (Berlin, Germany) under the proposal number EA4/085/21 and performed in accordance with the ethical standards as laid down in the 1964 Declaration of Helsinki and its later amendments. Informed consent was obtained from all subjects before study participation. Written consent from subjects and artists was obtained for the photographs of tattoos shown. Artist copyright was indicated as requested. The study was registered in the German Clinical Trials Register under DRKS00026022.

### Analysis of plasma, urine, tattoo inks and consumables

Blood samples were stored and transported at 2–8 °C before processing to plasma via centrifugation at 2500 rcf for 15 min (centrifuge 5804 R, Eppendorf, Hamburg, Germany). Due to canister size, urine samples were kept at room temperature by subjects and stored and transported at 2–8 °C upon arrival at the study centre. Urine volume was determined upon arrival at the BfR.

Quantification methods of all tracers were validated according to a guideline of the European Medicines Agency with additional parameters according to an ICH guideline (European Medicines Agency 2009; International Council for Harmonisation of Technical Requirements for Pharmaceuticals for Human Use 2019; Kochs et al. 2023).

For iodine quantification, plasma and urine samples as well as ink samples and consumables were analysed using inductively coupled plasma mass spectrometry (ICP-MS) after sample preparation as previously described (Kochs et al. 2023). The method is described in detail in *Supplemental Material 3*. Consumables were extracted with 1 l ultrapure water for 2 × 30 min on every side to extract iodide whilst shaking (250 rpm) before addition of the internal standard.

PABA, PEtOH and their metabolites were analysed in plasma, urine and ink using high-performance liquid chromatography coupled to a quadrupole time-of-flight mass spectrometer (HPLC-QTOF-MS) as previously described (Kochs et al. 2023). Isotope-labelled internal standards were utilised for quantification against a calibration curve in the corresponding matrix (plasma or urine). In brief, sample preparation included addition of internal standards, protein precipitation by ice-cold acetonitrile (Carl Roth GmbH & Co, Karlsruhe, Germany, catalogue number: HN40.2), centrifugation and further dilution with ultrapure water. In addition, alkaline hydrolysis was performed to determine the total PABA content in urine. For the analysis of PEtOH metabolite 2-phenoxyacetic acid (PAc) in urine samples, a standard addition method was used. The method is described in more detail in *Supplemental Material 3*. The cosmetic products used by the subjects were screened to confirm absence of PEtOH. No relevant concentrations were detected (*Supplemental Material 4*).

### Picture analysis

Digital photographs of the tattooed area were taken from a straight angle under appropriate light conditions. A measurement device was placed next to the tattoo for calibration purposes. Total tattooed body surface in cm^2^ was derived by digital picture analysis using the open-source software FiJi/ImageJ (version 2.3.0/1.53q) and its plugin Trainable Weka Segmentation. This method has already been previously applied for analysis of tattooed body surface (Foerster et al. 2023).

### Determination of tattoo ink use, urine retrieval and exposure

The amount of ink applied to the skin was calculated using two approaches (Fig. 1B,C). First, hypothetical use of ink per session (hypo) was derived by iodine quantification from consumables. The tattoo ink bottles were weighed before and after tattooing and the calculated amount of ink in the consumables was subtracted from the mass difference to obtain the amount of ink applied to the skin. Second, systemic exposure to ink components per session (sys) was calculated from the excreted amount of PABA or iodine in urine C-F. Since iodine has a physiological background and some individuals have a minor ACD background depending on their diet, urine A–B collected in the 24 h before tattooing was subtracted as subject specific background. Both, hypo and sys, were divided by the derived tattooed area from the picture analysis to obtain the amount of ink per cm^2^.

For the urine retrieval, the amount of each tracer excreted within 24 h (urine C–F) was divided by the expected amount of tracer in the ink per session (hypo). This is, to get a measure on the reliability of quantifying the applied ink by our method versus the actual systemic exposure. In addition, the mean retrieval of all tracers was determined for every subject.

### Data analysis

Graphs were created using GraphPad Prism Version 10.1.2 (GraphPad Software, Boston, MA, USA) and R (version 4.3.1). The standard deviation used in Microsoft Excel LTSC MSO (Redmond, WA, USA) was STDEV.P, as we aimed to display the deviation within our study population.

### PABA metabolism after oral uptake

To compare peroral and intradermal administration of substances by tattooing, three healthy volunteers took 50 mg of PABA supplement. Urine was collected according to the tattoo study (Fig. 1A) and analysed using the same HPLC-QTOF-MS method.

### PABA metabolism in cell culture

As PAAs and PABA can be metabolised by skin, particularly through *N-*acetylation (Bonifas and Bloemeke 2015; Eilstein et al. 2014), the metabolism of PABA was investigated in pooled human dermal fibroblasts (HDFp) from CELLnTEC advanced cell systems AG (Bern, Switzerland, catalogue number: HDfp, lot: MC1904099) and monocyte-derived macrophages (MDM) isolated from buffy coats of three different donors as previously described (Aparicio-Soto et al. 2020). The cultivation of cells is detailed in *Supplemental Material 5*. For the experiments, cells were seeded in 12-well plates (HDFp: 8 × 10^4^ cells/well and MDM: 8.75 × 10^5^ cells/well) and incubated for 24 h to reach a confluency of 70–80% before treatment with different concentrations of PABA (0, 0.1, 1, and 10 µg/ml) dissolved in Dulbecco’s modified Eagle’s medium/F12 1:1 (DMEM/F12) with L-glutamine, 1.2 g/l NaHCO_3_ but without phenol red from PAN-Biotech GmbH (Aidenbach, Germany, catalogue number: P04-41650). After another 24 h of incubation, the cell culture medium was collected and analysed using the above mentioned HPLC-QTOF-MS method.

## Results

### Quality control of study inks

All inks used in the study were analysed to confirm the absence of PEtOH. The concentrations of the tracers after their addition were determined and were all within a limit of ± 15% of the nominal tracer concentration. All analytical data, corrections (e.g. concentration of iodine in the ink had to be corrected for one subject and the concentrations of all tracers for one other subject), and justifications are reported in the supplements (*Supplemental Excel File*).

### Study subjects and deviations

The age of the subjects ranged from 22 to 43 years (median 32.5 years) and the body weight from 62 to 98 kg (median 80.5 kg). Height and body fat were 161 – 193 cm (median 181.5 cm) and 7.73 – 36.58% (median 18.38%), respectively. No dermatological observations other than normal skin reaction after tattooing were reported. Minor laboratory deviations were noted but judged by the study physician as clinically non-relevant. Other deviations included coffee consumption, intake of ibuprofen the day before tattooing, difficulties during blood sampling, deviations from the sampling times and the ink bottle falling over during tattooing. The spilled ink was cleaned up with wipes and sent in a separate bag which was analysed together with other consumables with ink residues. All subject data and deviations are reported in detail in the supplements (*Supplemental Excel File*).

### Ink exposure and urine retrieval of tracers

Data sets of two subjects (Black 9, Red 4) could not be fully included due to unsuccessful quantification of iodine in the consumables. They could, therefore, not be used to quantify the hypothetically applied ink per session (hypo). The PEtOH retrieval for subject Red 8 was 425.75%, and therefore significantly higher than the applied PEtOH concentration. It is possible that cosmetic products containing PEtOH were used during the study instead of the cosmetic products provided. However, the use of other cosmetic products was not reported. This value was, therefore, excluded from the calculations. It was also necessary to exclude PEtOH data of subject Black 12, since exclusion of several calibration points was necessary, leading to unreliable quantification (Table 1).

**Table 1:** Summary of study results. Total ink and related values are based on hypothetically (hypo) applied ink dependent on iodine quantification in consumables and ink weight

Subject	Session duration [min]	Tattooed area [cm^2^]	Ink per session (hypo) [g]	Ink per area (hypo) [mg_ink_/cm^2^]	Urine retrieval iodine [%]	Urine retrieval PABA [%]	Urine retrieval PEtOH [%]
Black 1	271	246.5	2.16	8.77	22.25	15.97	27.11
Black 2	206	62.0	0.92	14.90	33.08	33.62	40.43
Black 3	305	143.7	1.26	8.77	7.98	3.16	12.03
Black 4	255	185.2	1.25	6.73	9.37	6.46	8.41
Black 5	440	104.0	0.76	7.35	79.50	62.23	58.57
Black 6	340	62.0	0.74	11.85	26.97	18.12	19.62
Black 7	130	49.0	0.24	4.94	36.32	39.32	42.02
Black 8	200	80.0	1.61	20.09	32.86	20.14	22.71
Black 9	177	84.0	n/a	n/a	n/a	n/a	n/a
Black 10	277	150.5	1.29	8.60	31.48	24.52	23.19
Black 11	260	107.5	0.76	7.09	41.51	34.53	30.02
Black 12	171	90.5	0.25	2.73	20.17	17.10	11.65*
Black 13	231	63.5	1.38	21.72	17.31	20.94	3.76
Black 14	188	80.5	0.62	7.68	25.89	27.98	21.73
Red 1	119	59.0	1.17	19.81	10.93	7.06	3.49
Red 2	125	146.5	0.78	5.32	63.63	20.87	49.30
Red 3	210	22.0	0.87	39.45	8.36	7.51	4.60
Red 4	165	17.5	n/a	n/a	n/a	n/a	n/a
Red 5	155	41.0	1.20	29.27	38.44	23.22	24.53
Red 6	175	28.0	0.47	16.64	9.29	8.19	4.61
Red 7	120	80.0	0.86	10.76	24.78	27.75	29.40
Red 8	130	51.5	0.25	4.89	65.82	55.73	425.75*
Red 9	95	38.8	0.49	12.73	65.77	39.62	46.42
Red 10	160	80.6	0.30	3.71	53.78	55.37	59.48
Mean_all_	204.38	86.4	0.89	12.45	32.98	25.88	26.57
SD_all_	79.81	53.6	0.48	8.85	20.58	16.26	17.51
Median_all_	182.50	80.0	0.82	8.77	29.23	22.08	23.86
Mean_black_	246.50	107.8	1.02	10.09	29.59	24.93	25.80
SD_black_	76.98	53.7	0.52	5.42	17.31	14.72	14.77
Median_black_	255.00	87.3	0.92	8.60	26.97	20.94	22.71
Mean_red_	145.40	56.5	0.71	15.84	37.87	27.26	27.73
SD_red_	32.05	36.5	0.33	11.39	23.71	18.17	20.89
Median_red_	130.00	51.5	0.78	12.73	38.44	23.22	29.40

PABA = 4-aminobenzoic acid, PEtOH = 2-Phenoxyethanol. *Data were excluded from the overall evaluation

**Fig. 2:**
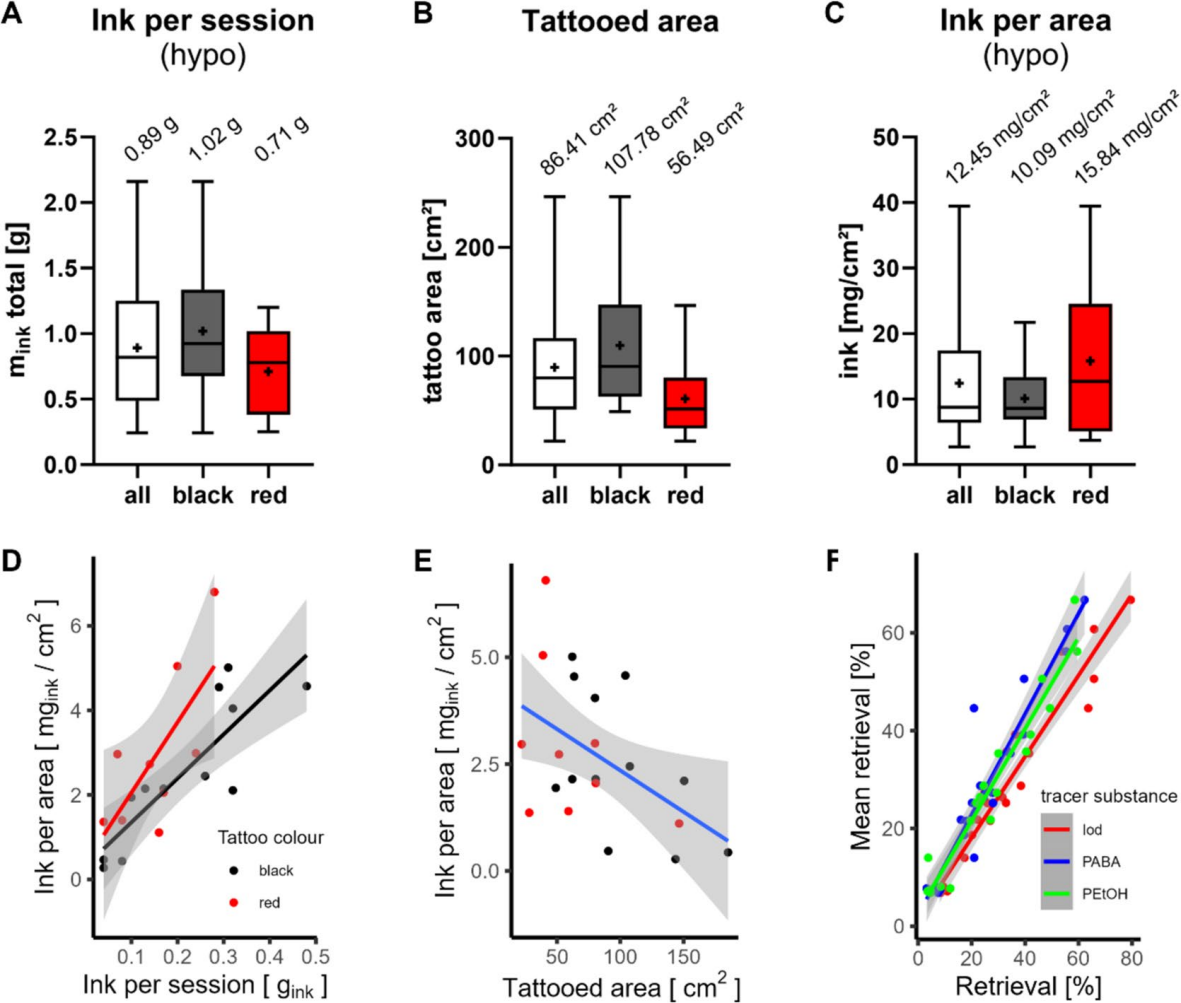
**A**–**C** Comparison of ink per session (hypo), tattooed skin area and ink per area of all subjects (n = 22), ink per session calculated from mass difference of ink bottle weight and subtracted ink residues from consumables. The box and whisker plots show median (line) and mean ( +) for all, black or red tattooed subjects. **D** The amount of tattoo ink per area is positively correlated with the ink use per session (systemic, sys, based on excretion of 4-aminobenzoic acid, PABA) with the slope being higher for red tattoos. **E** Ink per area (sys, PABA) is negatively correlated with the tattooed area. **F** Correlation plot of tracer retrieval to mean retrieval calculated for each subject. For **D**–**F**, regression line was calculated with a linear model (x ~ y) and the 95% confidence interval was plotted in grey

The average ink quantity per session (hypo) was 0.89 ± 0.48 g for both colours combined, 1.02 ± 0.52 g for black and 0.71 ± 0.33 g for red (Table 1*, *Fig. 2A). The tattooed areas were subjectively estimated to be around 300 cm^2^ for black and 100–300 cm^2^ for red, whereby non-tattooed, empty spaces were included in the area. This was a prerequisite in subject recruitment, since we aimed for larger tattoos to reach detectable amounts of tracers in blood and urine. However, as demonstrated by Foerster et al. (2023) the actual tattooed areas are often smaller than the estimated areas. We, therefore, used the same picture analysis method to calculate the area of the tattooed surface. The analysis of the tattooed area per session resulted in an average size of 86.4 ± 53.6 cm^2^ for both colours, with the average being 107.8 ± 53.7 cm^2^ for black tattoos and 56.5 ± 36.5 cm^2^ for red tattoos (Table 1*, *Fig. 2B). The estimated exposure to tattoo ink per skin area (hypo) was, therefore, 12.45 ± 8.85 mg_ink_/cm^2^, with 10.09 ± 5.42 mg_ink_/cm^2^ for black and 15.84 ± 11.39 mg_ink_/cm^2^ for red (Table 1*, *Fig. 2C).

**Fig. 3:**
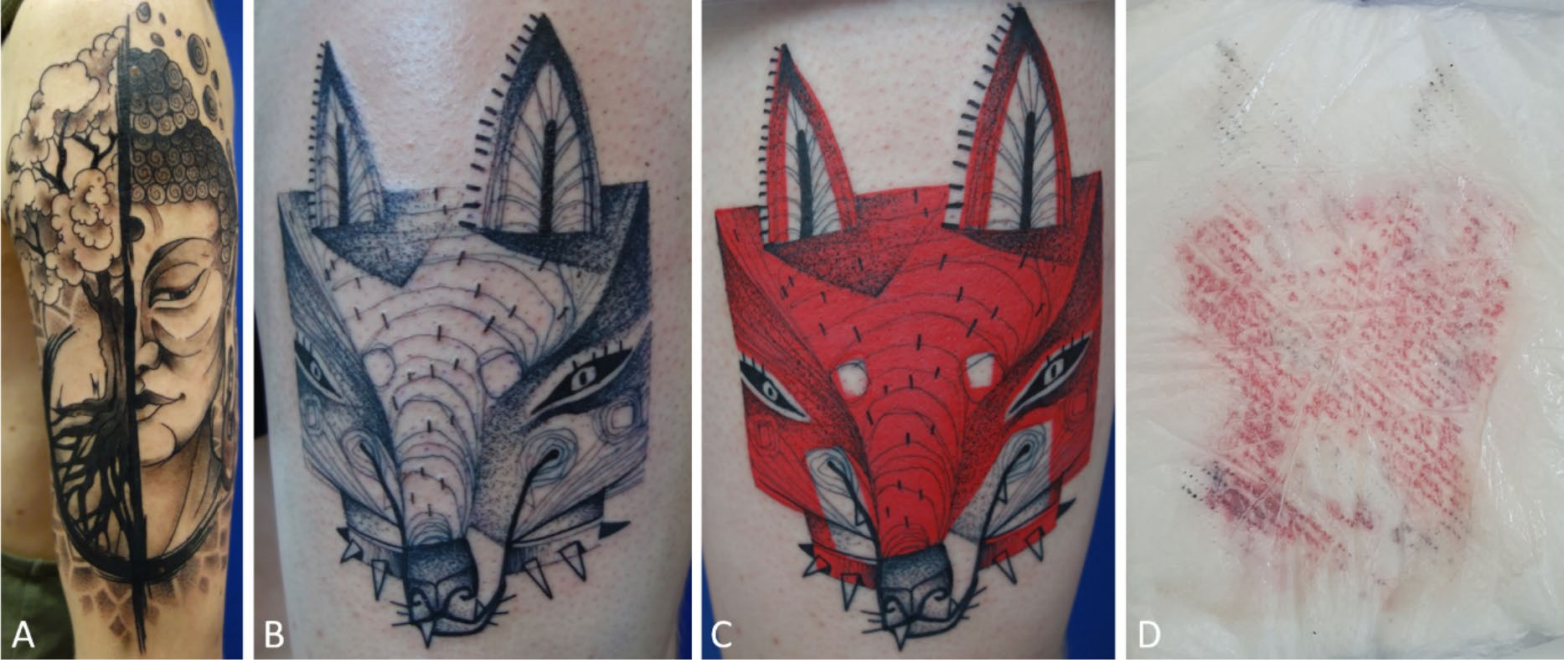
Pictures of tattoos from the study. **A** Black tattoo of subject Black 5 with the highest urine retrieval. **B** The black outlines of subject Red 9 were tattooed beforehand and did not contain any tracers. **C** Tattoo in (**B**) with red areas. Blood samples were drawn shortly before and at specific time points after the start of tattooing with red ink containing the tracers. **D** Wound dressing of a red study subject displays exudation of black and red tattoo ink within the first hours after tattooing. © Artist: Alexander Pietsch (A), Carlo Sohl (B,C)

Black ink was used for contouring, shading and for monochromatic tattoos, whereas red ink was often used to fill larger continuous areas (Fig. 3A–C). In some cases, outlines or other shades were done before start of tattooing with spiked ink (Fig. 3B). The ink used for this purpose did not contain tracers and the start of blood sampling was aligned with the start of tattooing with spiked ink (Fig. 3C). Tattoo aftercare varied between tattoo artists. In one case, a wound dressing was applied to a red study subject and displayed exudation of black and red ink (Fig. 3D).

### Adjustments for absorbed fraction of tracers

The retrieval of all three tracers after 24 h was calculated by dividing the sum of excretion of each tracer in urine C–F by the initial amount of tracer in the ink per session (hypo). Although the average retrieval for all three tracers within each individual study subject were similar, the tracer retrievals for all 24 subjects were unexpectedly low and varied between 3.16 and 79.50% (Table 1*, *Fig. 2F). Therefore, we re-evaluated the data with different approaches.

First, the data set was reduced by excluding subjects with a mean urine retrieval of < 25% (n = 13), since extremely low retrievals are most likely error prone (Fig. S1*A–C*). The average amount of ink applied was 0.78 g for the reduced data set, and therefore lower compared to the full dataset (Fig. 2A–C) with 0.89 g of ink. Overall, the data scattering was lower for the reduced data set.

Second, peroral dosing of PABA showed complete retrieval in urine within 24 h (94.75 ± 3.54%, cf. section *PABA metabolism after oral and intradermal administration*). Since PABA is fully dissolved in the tattoo ink, it can be assumed that it is freely available in the body, as was the case in peroral administration. Therefore, the sum of excreted PABA in urine corresponds to the systemically available fraction of the applied ink after tattooing. Hence, the applied ink per session (sys) was calculated from PABA retrieved within 24 h after the start of tattooing (Table 2). Similarly, applied ink was approximated by iodine excretion with subtraction of the iodine background from the 24 h before tattooing (Fig. S1*D–G*). For both iodine and PABA, the estimation of ink per session (sys) was about 25% of the hypothetically applied ink (hypo) after quantification via consumables.

**Table 2:** Comparison of mean ink exposure calculated from study data and its 75th percentile with data used in the Annex XV restriction report

Basis of calculation	Sample size	Ink per session [g]		Ink per area [mg_ink_/cm^2^]	
		Mean	75th p.	Mean	75th p.
Ink per session (hypo)	n = 22	0.89	1.25	12.45	17.44
	(n = 13, retrieval > 25%)	0.78	1.06	10.56	13.82
Ink per session (sys, PABA)	n = 24	0.19	0.29	2.62	3.78
Ink per session (sys, iodine)	n = 24	0.25	0.39	3.37	3.67
Mean ink per session (sys, PABA/iodine)	n = 48	0.22	0.31	2.99	3.67
Annex XV restriction report	n = 9		4.31		14.36

75th p. = 75th percentile, PABA = 4-aminobenzoic acid, sys = systemic available ink, hypo = hypothetically applied ink

The slope of the correlation of ink per area with ink per session (sys, PABA) is higher for red tattoos than black tattoos (Fig. 2D). Likewise, a negative correlation with tattooed area was demonstrated for both colours (Fig. 2E). An additional multiple factor analysis showed a minor connection between ink per area (sys, PABA), urine excretion and body fat (*Supplemental Material 6*).

In the Annex XV restriction report, the 75th percentile of previously available data was used to calculate the ink per area, which resulted in an estimation of 14.36 mg_ink_/cm^2^ (European Chemicals Agency 2017). The use of the 75th percentile was justified by the REACH guidance documents R15 on consumer exposure and R14 on occupational exposure, where a deviation from the 90th percentile can be justified if a worst case, such as limited data for assessment, occurs (European Chemicals Agency 2016a, 2016b). Thus, we also applied the 75th percentile to our data set (Table 2). On the basis of the ink per session (hypo), our 13.82 mg_ink_/cm^2^ for the reduced data set with a retrieval > 25% were close to the Annex XV restriction report estimations (European Chemicals Agency 2017). However, when ink per session (sys) was applied, the mean 75th percentile was 3.67 mg_ink_/cm^2^ (Table 2).

### Metabolite profile and plasma kinetics

**Fig. 4:**
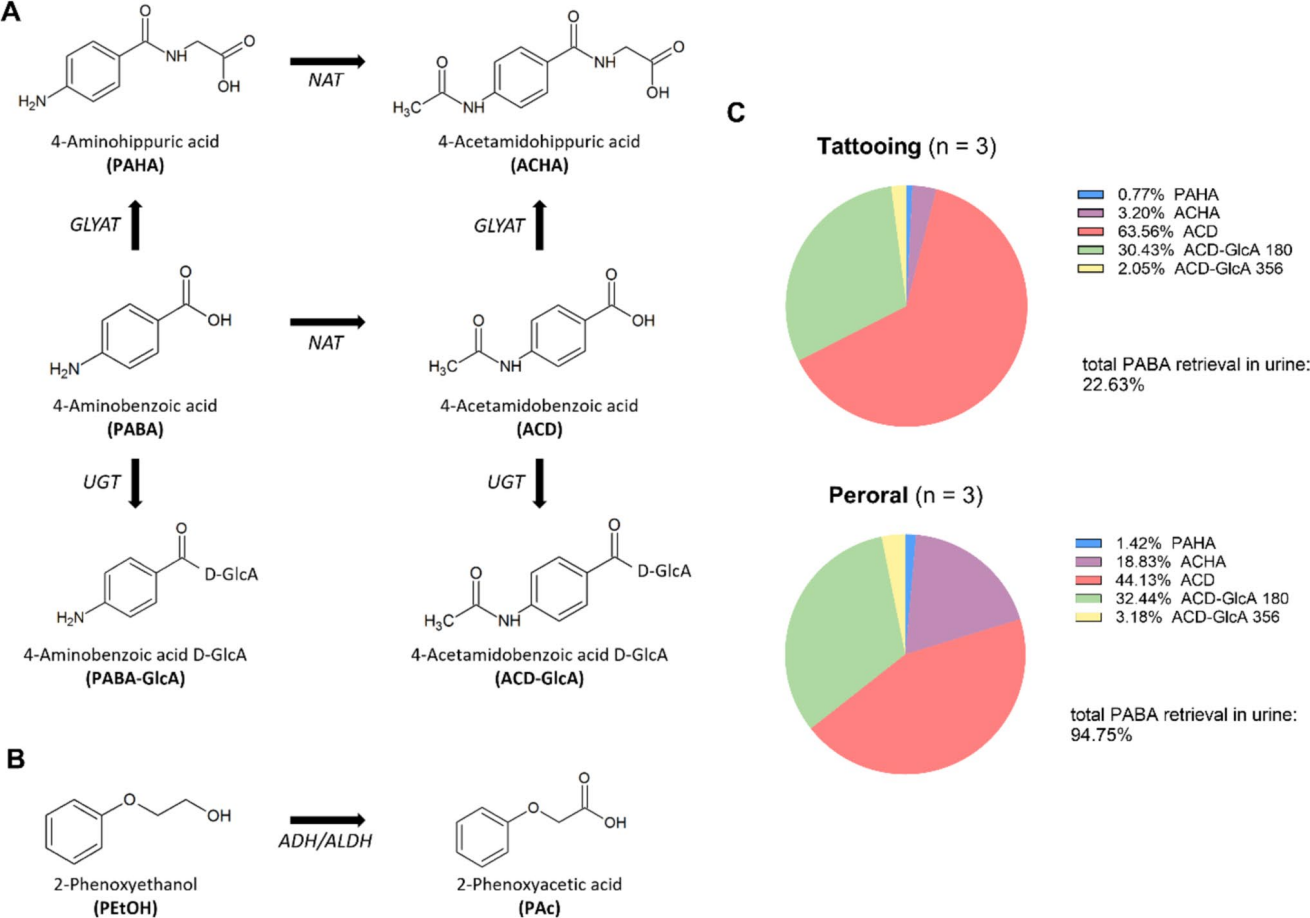
Analysis of 4-aminobenzoic acid (PABA), 2-phenoxyethanol (PEtOH) and their metabolites. **A** Metabolism of PABA. GLYAT: glycine-*N*-acyltransferase. NAT: *N*-acetyltransferase. UGT: UDP-glucuronosyltransferase. **B** Metabolism of PEtOH. ADH: Alcohol dehydrogenase. ALDH: aldehyde dehydrogenase. **C** Comparison of the metabolite distributions in total urine (urine C–F) calculated from raw peak area ratios. Data are displayed for three tattoo study subjects (Black 8, Black 10, Red 5) and the three peroral PABA subjects for the first 24 h after tattooing

Several PABA metabolites were identified in the subjects’ urine (Fig. 4A). ACD was quantified in all subjects, whereas PAHA was only above the limit of detection (LOD) in 15 subjects (*Supplemental Excel File*). The quantifiable metabolites of PABA were predominantly in the form of ACD (41.76 ± 11.15%), with only a small percentage in the form of PAHA (0.79 ± 0.76%) (Fig. S2). In addition, metabolites that could not be quantified directly, such as ACD-GlcA and ACHA, were detected based on their monoisotopic masses (355.09 g/mol for ACD-GlcA and 236.08 g/mol for ACHA) and the corresponding extracted ion chromatograms (Fig. S3). PEtOH was only detected in form of metabolite PAc in urine samples (Fig. 4B*, Supplemental Excel File*).

In plasma, iodine and PABA metabolite ACD were detected; however, ACD concentrations were close or below the limit of quantification (LOQ) or LOD (*Supplemental Excel File, Fig. S4)*. This was particularly the case for subjects with low total PABA excretion in urine.

### PABA metabolism after oral and intradermal administration

Peroral administration to three individuals resulted in an average PABA urine retrieval of 94.75 ± 3.54% (Table S1), which is considered as complete excretion. The metabolic profile of all three individuals was rather similar indicating low inter-person variability. For comparison between peroral and intradermal administration, data of three tattoo study subjects were selected based on their similar applied ink per session (hypo), resulting in a similar applied amount of PABA (48.21 mg, 36.00 mg, 38.82 mg). Also, their total PABA retrieval in urine of 22.63 ± 1.84% was similar (Table S1).

As some metabolites could not be quantified, a peak area comparison was carried out. Peak areas are not necessarily related to concentrations since ionisation efficiencies of each metabolite may vary significantly. However, it allows for a general comparison of the metabolite profile of the two routes of administration. Therefore, all corresponding peaks were integrated and the ratio of the respective peaks to the total peak area of all metabolites in urine C–F was determined (Fig. 4C). After 24 h, almost one-fifth (18.83 ± 3.26%) of the peak areas correspond to ACHA when administered perorally, whereas intradermal application resulted in 3.20 ± 0.69%. The ACD content was higher after tattooing compared to peroral administration, which was already determined by quantification. No differences were observed for the ACD-GlcA areas.

### PABA metabolism in fibroblasts and monocyte-derived macrophages

Since our study results and the peroral comparison indicate a cutaneous first-pass effect during tattooing, the metabolic capacity to form ACD in tattoo-relevant skin cells was investigated. After 24 h of incubation with PABA, both fibroblasts and monocyte-derived macrophages catalysed formation of ACD (Table S2). The samples were also screened for other known PABA metabolites via the corresponding extracted ion chromatograms in the obtained data sets, but no other metabolites were observed.

### Discussion

In this study, we obtained in vivo human exposure data on tattooing that are the first of their kind and represent a reasonable worst-case scenario. These data can be used to adapt risk assessments related to carcinogens and other toxic substances in tattoo and permanent make-up inks, thereby allowing for a better prediction of tattoo-associated health risks in the general population. In addition, our data provide evidence for the capacity of skin cells to metabolise certain substances to an extent that affects parent compound-inherent toxic properties during tattooing, especially in the case of PAAs. A limitation of our study is that an unknown amount of ink components seems to be lost via wound healing, which should be studied further.

Iodine quantification in plasma was most successful since it is least affected by matrix effects and has a significantly lower LOD (Kochs et al. 2023). Neither PEtOH nor its metabolite PAc were detected in plasma. Given that the concentration of the PABA metabolite ACD was already close to the LOD, it is reasonable that neither PEtOH nor PAc could be detected, as the PEtOH concentration in the ink was six times lower when compared to PABA and detection limits of PEtOH and PAc were slightly higher (Kochs et al. 2023). Blood samples only reflect a specific time point, bearing the risk to miss the peak concentrations or any concentration above the LOD within the plasma kinetics. In urine samples, both PABA and PEtOH parent compounds were absent since they undergo fast metabolic conversion. Also, PABA-GlcA was not found despite previously reported as PABA metabolite (Chan et al. 1988). In case of PEtOH, in silico (Hewitt et al. 2022) and animal experiments (Hartwig and MAK Commission 2017; Scientific Comittee on Cosumer Safety 2016) suggest a variety of possible metabolites, including those formed via oxidation, hydroxylation, sulfation or glucuronidation. In one human study, PEtOH was excreted in the form of both PAc (85%) and PAc conjugates (15%, n = 1), whereas another study on four subjects only detected PAc (Hartwig and MAK Commission 2017; Scientific Comittee on Cosumer Safety 2016). A recent study by Eckert et al. identified 4-hydroxyphenoxyacetic acid as an additional metabolite in a lower ratio compared to PAc (Eckert et al. 2024). In our study, no metabolite other than PAc was found. However, the PEtOH concentration used was much lower compared to the study by Eckert et al., hence it may be possible that 4-hydroxyphenoxyacetic acid was formed but below identification limits.

In our study, red tattoos correlated with higher use of ink but smaller tattooed areas when compared to black tattoos. This correlation can be explained by the fact that red tattoo designs mostly consisted of filled areas rather than shading and outlines which were more prominent in black tattoos. In the picture analysis, shaded areas were not considered. Therefore, tattoo design rather than ink colour plays an important role in the degree of ink exposure. Minor correlations of ink per area and total excreted urine volume were observed. With the small number of study subjects, the power of such analysis is limited. However, similar findings regarding increased excretion of iodine and other biomarkers with high urine volume have been reported previously (Johner et al. 2010; Pinto et al. 2022).

A subject’s wound dressing showed distinguishable red and black tattoo ink remains after 24 h, indicating ink exudation through the damaged skin barrier*.* It is, therefore, most likely that not all of the ink applied will be absorbed by the body. Also, the retrieval of tracers was extremely variable and ranged between 3.16 and 79.50%. Since all three tracers resulted in similar retrievals within each subject, analytical errors are unlikely to cause the low tracer retrieval. An important source of errors is the indirect calculation of ink in consumables. Failing to collect all materials could lead to an underestimation of ink in consumables, leading to an overestimation of applied ink. Due to the variety of materials used by the artists that were not all tested during method validation, quantitation errors of the amount of ink adhered to consumables are certainly possible. Incomplete urine collection could also lead to an underestimation of excreted tracers. Factors influencing the absorption of ink into the body may include skin histology of different body parts, the tattoo artist’s technique or the wound healing process. Compound retention within the skin or the lymphatic system leading to a depot effect does not seem plausible in our view. Since the retrieval rates in urine were similar for all three tracers, a possible depot effect in tissue could be only explained by physico-chemical differences, but is less likely to occur with small molecules (or ions) completely dissolved in the application media (Benedetti et al. 2009). Therefore, the most plausible factor responsible for the discrepancies in the systemic exposure levels might be transepidermal loss by wound healing. Accordingly, only a fraction of the hypothetically applied ink actually entered the body and that the fraction of soluble ink components absorbed into the body is likely overestimated using the hypothetically applied ink for exposure calculations.

This conclusion is also substantiated by our peroral in vivo data. PABA was completely excreted within 24 h in the peroral setup, whereas PABA retrieval during tattooing was only about 25% of the hypothetically applied ink. We concluded that the absorbed fraction of PABA and its metabolites would also be fully excreted during tattooing and calculated the systemically absorbed ink fraction from total PABA (and iodine) retrieved in urine. Iodine was included because retrieval results were similar to those of PABA. In this case, inaccuracies in consumable collection and its iodine quantification are not relevant. Also, PABA is mainly excreted within the first hours after tattooing where study participants were still at the study centre, and where compliance in terms of complete urine collection is more reliable than at home. Therefore, only little effects on the exposure estimation are expected.

In the Annex XV restriction report, the 75th percentile of the available data set was used to calculate a reasonable worst-case scenario, as these data reflected a high exposure situation only (European Chemicals Agency 2017). In this study, only medium to large tattoos were included. Hence, according to our data, the 75th percentile of 0.31 g ink per session (sys) displays a reasonable worst-case exposure level per tattoo session. This value is about 14-times lower than the 4.31 g per session previously estimated. Applying the exposure levels calculated from our data would have a significant impact on the risk assessment and health risk predictions of hazardous substances. For example, a market survey from 2022 analysed tattoo inks for their preservative concentrations and the highest PEtOH concentration found was 6475 µg/g_ink_ (about 0.65% PEtOH)(Famele et al. 2022). Assuming that our calculated exposure value of 0.31 g ink per session is accurate, approximately 2 mg of PEtOH are introduced into the body during one tattooing session. However, according to REACH, only 0.01% PEtOH are allowed in tattoo inks due to its eye irritation properties. In cosmetics, 1% of PEtOH can be used and 0.5% is a common concentration to preserve injected pharmaceuticals (Meyer et al. 2007). One of the highest findings of PAAs was 1775 µg/g_ink_ *o*-anisidine pre-REACH (Fels et al. 2023). However, PAA limits set by REACH are concentrations up to 5 µg/g_ink_. Here, a value of 0.31 g of ink would result in a maximum of 1.55 µg PAA entering the body when applying a REACH compliant tattoo ink preparation under reasonable worst-case conditions.

The majority of research to date has been conducted in Europe and tattoo inks are currently more regulated in the European Union than in other regions. The United States are also seeking to increase consumer protection with the Modernization of Cosmetics Regulation Act of 2022 (MoCRA), which would also apply to tattoo inks (U.S. Food and Drug Administration 2022). However, no quantitative limits for toxic substances in tattoo inks are defined in the proposed legislation.

In general, toxicological data on the intradermal application of substances are scarce. Therefore, transfer of data from other routes of administration (e.g. peroral) to the intradermal application route is justified to obtain an idea of the level of internal exposure. An important factor distinguishing tattooing from other absorption routes (e.g. peroral or intravenous) is cutaneous metabolism. Therefore, we compared the peroral intake of the tracer PABA (n = 3) to absorption via tattooing and investigated the metabolism of PABA in tattoo-relevant cells. The normalised peak area of the metabolite ACHA was more dominant in peroral administration when compared to intradermal administration, possibly due to the hepatic and gastrointestinal first-pass effect. No peak in the extracted ion chromatograms of PABA-GlcA was detected during chemical analysis, indicating that either this is not a favoured metabolic pathway at the concentrations administered in this study or that its signal was below background signal. When normalised to the total hydrolysed PABA in urine, the tattoo samples contained about twice as much ACD compared to peroral intake. Therefore, skin metabolism may play a role in detoxification of substances in inks that are known targets of the enzyme *N*-acetyltransferase 1. A detoxifying first-pass effect by *N*-acetylation of a genotoxic PAAs (i.e. 2,4-toluenediamine) in skin and different skin cell types was previously described (Grohmann et al. 2017). Keratinocytes in the epidermis, which are known for their capacity to catalyse *N*-acetylation (Bonifas and Bloemeke 2015; Eilstein et al. 2014; Hu et al. 2010; Oesch et al. 2007), may be in contact with the ink constituents during tattooing and in the early phase of wound healing. In addition, fibroblasts and macrophages might also partake in ACD formation during tattooing. The comparison of peroral with intradermal and cell culture data indicates that the substances are likely to be metabolised in the skin during tattooing. A major difference between the two routes of administration is that tattooing delivers the dose unevenly over a longer period of time, depending on the motif, whereas peroral administration delivers the dose all at once. These differences certainly have an effect on the ratio of metabolites, as the routes of metabolism often depend on the dose of substance reaching the tissue. The quantitative impact on the detoxification of aromatic amines by this cutaneous first-pass effect compared to peroral administration is yet to be understood. Kinetic modelling might help to address this data gap in the future.

In conclusion, the present study fills the data gaps on systemic exposure to tattoo ink ingredients needed for health risk assessments. Given the loss of soluble and insoluble ink components via wound healing, future studies particularly should consider exudation. The data presented can be used to simulate the kinetics and internal exposure to these substances—ideally taking into account the cutaneous first-pass effect during tattooing as shown in this study. This may help to predict kinetics of substances that cannot be tested in human subjects due to their toxicological profile and to extrapolate toxicity data obtained from other sources (e.g. oral animal or in vitro data). Such additional data will contribute significantly to the efforts of our study to translate the kinetics and exposure data into health protection for consumers. The study data presented here should be used for discussion with competent authorities to develop harmonised exposure assessments and to evaluate tattoo-associated risks in the future.

## Supplementary Information

Below is the link to the electronic supplementary material.

Supplementary file1 (XLSX 80 KB)

Supplementary file2 (DOCX 356 KB)

Supplementary file3 (PDF 594 KB)

## Data Availability

All data generated or analysed during this study are included in this published article. The raw data generated during and/or analysed during the current study are available from the corresponding author on reasonable request. Sensitive information in the form of confidential or personal data is excluded.
